# Lab indicators standardization method for the regional healthcare platform: a case study on heart failure

**DOI:** 10.1186/s12911-020-01324-6

**Published:** 2020-12-15

**Authors:** Ming Liang, ZhiXing Zhang, JiaYing Zhang, Tong Ruan, Qi Ye, Ping He

**Affiliations:** 1 School of Information Science and Engineering, East China University of Science and Technology, 130 Meilong Road, Shanghai, 200237 China; 2 Shanghai Hospital Development Center, 2 Kangding Road, Shanghai, 200000 China

**Keywords:** Lab indicator standardization, Entity alignment, Active learning, Machine learning, Electronic health record, Heart failure

## Abstract

**Background:**

Laboratory indicator test results in electronic health records have been applied to many clinical big data analysis. However, it is quite common that the same laboratory examination item (i.e., lab indicator) is presented using different names in Chinese due to the translation problem and the habit problem of various hospitals, which results in distortion of analysis results.

**Methods:**

A framework with a recall model and a binary classification model is proposed, which could reduce the alignment scale and improve the accuracy of lab indicator normalization. To reduce alignment scale, tf-idf is used for candidate selection. To assure the accuracy of output, we utilize enhanced sequential inference model for binary classification. And active learning is applied with a selection strategy which is proposed for reducing annotation cost.

**Results:**

Since our indicator standardization method mainly focuses on Chinese indicator inconsistency, we perform our experiment on Shanghai Hospital Development Center and select clinical data from 8 hospitals. The method achieves a F1-score 92.08$\%$ in our final binary classification. As for active learning, the new strategy proposed performs better than random baseline and could outperform the result trained on full data with only 43$\%$ training data. A case study on heart failure clinic analysis conducted on the sub-dataset collected from SHDC shows that our proposed method is practical in the application with good performance.

**Conclusion:**

This work demonstrates that the structure we proposed can be effectively applied to lab indicator normalization. And active learning is also suitable for this task for cost reduction. Such a method is also valuable in data cleaning, data mining, text extracting and entity alignment.

## Background

Electronic health records (EHRs) have been applied to many clinical data analysis, such as prognostic analysis and decision support. In EHRs, laboratory indicator test results are considered to be important factors. For example, “  ” (Aspartate aminotransferase, AST) can be regarded as a diagnostic and prognostic indicator of myocardial infarction [1]. However, many same indicators are presented using different names in Chinese. There may be two main reasons: The first one is a translation problem. Aspartate aminotransferase can be translated as “  ”, “  ”, “  ”, “  ”, etc, in Chinese. The second one is a habit problem. Different hospitals or different doctors prefer different names or have their own statements, such as “  ”, “  ” and “  ”. Since there exists no such a synonym knowledge base (KB), EHRs associated with these synonymous names will be lost, resulting in distortion of the analysis results. Therefore, it is necessary to design an automatic method for lab indicators standardization.

An effective way to solve the standardization of lab indicators problem is entity alignment. There are two main tasks related to the entity alignment: instance matching and entity linking. Instance matching [2] aligns same entities in different KBs. A lot of traditional methods are based on feature engineering [3, 4], and utilize attribute values [5, 6], structural information [7], external lexicons [8–10] and so on. Recent methods are based on representation learning [11–16], and embed entities into vectors for similarity calculation. Entity linking aligns a mention in a text and an entity in a KB. It has both unsupervised methods [17–19] and supervised methods [20–23]. However, there is no attribute or context in our data, these two tasks cannot apply to our standardization directly. Zhang et al. [24] proposed a n-gram and stacking enhanced method based on indicator name, abbreviation and reference value, which has a lot of space to improve because they only use handcraft feature. Besides, all the methods mentioned above ignore the cost of annotations. Unlike the tasks in universal corpus, indicator normalization requires experienced domain experts for annotating. Active learning aims to select few samples for training without degrading much performance [25, 26]. And it has been successfully applied in many tasks [27–29]. So the annotation cost is also considered in our work.

In this paper, a framework is used for indicator normalization. Including a recall model and a binary classification model, and then is evaluated by a case study on heart failure, which extends our previous work [24]. The main contributions of our work are as follows.

- An effective framework is proposed to normalize the lab indicators, which combines a recall model and a binary classification model. The purpose of the recall model is to reduce alignment scale, a candidate set contains standard indicators is selected for each non-standard indicators. After this step, candidate-standard indicator pairs can be generated by a binary classification model through an enhanced sequential inference model(ESIM) based on the name and abbreviation of indicators. Experimental results of the proposed structure show that it achieves an F1-score of 92.08$\%$ in the final binary classification.
- Active learning is utilized for reducing annotation cost. A new selection strategy is proposed and is compared with shannon entropy, least confidence and a random baseline. Experiments show that the our strategy performs better than random baseline and could outperform the same result which is trained on full data with only 43$\%$ training data.
- A detailed case study on heart failure clinic analysis is conducted on the sub-dataset from the dataset of a regional healthcare platform called Shanghai Hospital Development Center (SHDC). The result shows that our proposed method is practical in data cleaning, data mining, text extracting and entity alignment.

## Methods

**Fig. 1:**
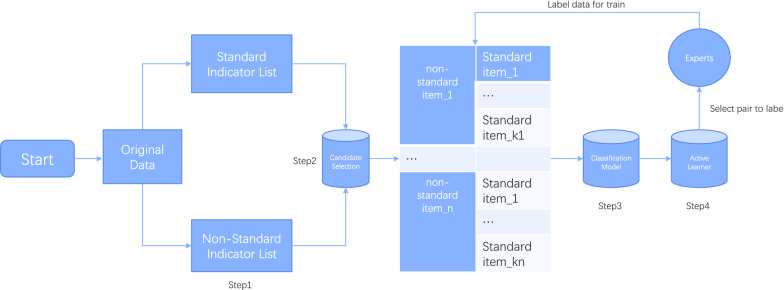
The overall process of the indicator standardization algorithm

Figure 1 shows the framework of the whole process, which include four parts. The first part is data pre-processing. With the help of experienced experts, a list of standard indicators is selected and a set of non-standard indicators is remained. A recall model is then presented in second part. For each non-standard indicators, a standard indicator candidate set is generated to reduce the alignment scale. The third part is a binary classification model to determine whether a non-standard indicator is synonymous with a standard indicator. Finally, active learning is applied to decline the cost of annotation.

### Data pre-processing

The original data we collected from hospital is a set of indicators with name and abbreviation. The aim of data pre-processing is to generate a standard indicator set. Though terminology standard like LOINC has been published early, it has not been widely used in clinical practice. In Zhang’s work [24], lab indicators are firstly clustered and then the most frequent term in each cluster is picked as the standard terminology. But we think that the set of standard indicator matters in indicator normalization. So we invite experts to select a list of standard indicators $S = (s^{1}, s^{2},\ldots ,s^{m})$, in which $s^i = (s_{n}^{i}, s_{a}^{i})$. The remaining set $L = (l^{1}, l^{2},\ldots ,l^{n})$ are regarded as a non-standard indicator collection, in which $l^{i} = (l_{n}^{1}, l_{a}^{1})$, subscript *n* and *a* represent name and abbreviations, respectively.

### Candidate selection

We collect a standard indicator set containing 1970 items through the first step. For a single non-standard indicator, it would be costly if it is compared with each item in standard indicator set due to the huge amount of standard indicator list. Similar challenges occur in recommending system and query engine, where a light recall model is often used to shrink the scale of candidate set, then a complicated model is applied for further processing. We use the similar idea in our task. To improve the efficiency of the whole system, a recall model is used to compute the similarity between non-standard and standard indicators. Each indicator has two attributes: name and abbreviation value. So two candidate set $C_{n}^{i} = (l_{n}^{i}, c_{n}^{i}), (l_{n}^{i}, c_{n}^{i}),\ldots ,(l_{n}^{i}, c_{n}^{k_{1}})$ and $C_{a}^{i} = (l_{a}^{i}, c_{a}^{i}), (l_{a}^{i}, c_{a}^{i}),\ldots ,(l_{a}^{i}, c_{a}^{k_{2}})$ is then generated for each non-standard indicator, in which $c_{n}^{i},c_{a}^{i} \in S$. Finally, we choose the union of $C_{n}^{i}$ and $C_{a}^{i}$ as the candidate set of $l^{i}$, notated as $C^{i}$.

The recall model selects a certain amount of candidates in a short time with a high recall rate. In this task, the textual similarity between the names and abbreviations of non-standard indicators and standard indicators is very high. So we have tried four classical text matching model: edit-distance, bow(bag of words), bm-25 and tf-idf. Experiments show that tf-idf works best on the test dataset. Tf-idf is described as follow. For a document set $D = \{d_1, d_2, \ldots d_n\}$, each document is composed by a list of words $\{w_1, w_2, \ldots , w_l\}$. Formula 1–3 shows the tf-idf value of $w_j$ in $d_j$, in which $f(w_j, d_i)$ is the frequency of $w_j$ in $d_i$ and $N_{w_j}$ is the number of documents where $w_j$ appears. The name or abbreviation of each indicator can be represented by a vector *v* whose length is equal to the length of the vocabulary. The corresponding value of each word in *v* is the tf-idf value of the word. Then the similarity between two indicators is represented by the doc product of their corresponding vector. The abbreviation of some indicator is missing during practice, so the length of candidate set for each non-standard indicator is unequal, range from $k_1$ to $k_1 + k_2$.

1 $$\begin{aligned}&tf\text {-}idf(d_i, w_j) = tf(w_j) * idf(w_j) \end{aligned}$$

2 $$\begin{aligned}&tf(d_i, w_j) = \frac{f(w_j, d_i)}{|d_i|} \end{aligned}$$

3 $$\begin{aligned}&idf(d_i, w_j) = log(\frac{|D|}{N_{w_j}+1}) \end{aligned}$$

### Binary classification

Considering that the recall model has sharply decreased the amount of candidates for each non-standard indicator, we apply a binary classification model to judge the relationship between a non-standard indicator and its candidate standard indicators. Before training the model, we combine the name and abbreviation of each indicator. For example, an indicator with name “  ” and abbreviation “ph” will be transferred to “  ”, which avoiding training two model targeted to name and abbreviation separately. We note the non-standard indicator and the standard indicator as query and hypothesis, represented as *a* and *b* respectively.

We define the binary classification as a natural language inference task. Though pretrained language model such as Bert [30] achieves state of the art in many domains and tasks, models based on lstm or cnn require less resource without degrading much performance. Chen et al. [31] proposed ESIM for text matching, which extract features of query and hypothesis by lstm and utilizing attention mechanism to interact query with its counterparts. Based on the reason mentioned above, we choose ESIM for binary classification.

ESIM consists of four parts. Input encoding utilize BiLSTM to represent each word with its context. Local inference modeling apply attention mechanism to interact query with hypothesis. Inference composition is proposed to compose the output of previous layer into a fixed length vector. Finally, a prediction layer put the vector into a multilayer perceptron classifier for classification.

#### Input encoding

For input *a* and *b*, BiLSTM is used to learn a representation of a word and its context, notated as ${\hat{a}}$ and ${\hat{b}}$ in Eqs. 4–5. ${\hat{a}}_i$ represent the hidden state generated by the BiLSTM at time *i* on the input sequence, which is same with ${\hat{b}}_i$.

4 $$\begin{aligned} \hat{a_i}= & {} BiLSTM_{1}(a_i), \forall {i \in {[1,\ldots ,len(a)]}} \end{aligned}$$

5 $$\begin{aligned} \hat{b_i}= & {} BiLSTM_{2}(b_i), \forall {i \in {[1,\ldots ,len(b)]}} \end{aligned}$$

#### Local inference modeling

Local inference modelling apply attention mechanism to capture the local reference between query and hypothesis, which is formulated in Eqs. 6–10. The attention score $e_{ij}$ is the doc product of $\hat{a_i}$ and $\hat{b_j}$. The alignment representation $\tilde{a_i}$ is then composed by ${\hat{b}}$, which taken $e_{ij}$ as weight. To get an enhanced representation, the element-wise product and the difference of ${\hat{a}}$ and ${\tilde{a}}$ is calculated, and concatenate with ${\hat{a}}$ and ${\tilde{a}}$ to get $m_a$ and $m_b$.

6 $$\begin{aligned} e_{ij}= & {} \hat{a_i}\hat{b_j} \end{aligned}$$

7 $$\begin{aligned} \tilde{a_i}= & {} \sum _{j=1}^{\ell _b} \frac{\exp (e_{ij})}{\sum _{j=1}^{\ell _b}\exp {e_{ij}}}\hat{b_j}, \forall i \in [1,\ldots \ell {a}] \end{aligned}$$

8 $$\begin{aligned} \tilde{b_i}= & {} \sum _{j=1}^{\ell _a} \frac{\exp (e_{ij})}{\sum _{j=1}^{\ell _a}\exp {e_{ij}}}\hat{a_j}, \forall i \in [1,\ldots \ell _{b}] \end{aligned}$$

9 $$\begin{aligned} m_a= & {} [{\hat{a}}, {\tilde{a}}, {\hat{a}} - {\tilde{a}}, {\hat{a}} \odot {\tilde{a}}] \end{aligned}$$

10 $$\begin{aligned} m_b= & {} [{\hat{b}}, {\tilde{b}}, {\hat{b}} - {\tilde{b}}, {\hat{b}} \odot {\tilde{b}}] \end{aligned}$$

#### Inference composition

Before the final prediction, a composition layer is proposed to compose the previous enhanced representation. Then a pooling layer is applied to convert the resulting vector obtained above to a fixed-length vector. Finally, the fixed-length vector is put into a multilayer perceptron classifier taken softmax as activation function. Cross-entropy is used for training.

11 $$\begin{aligned} v_{a,i}= & {} BiLSTM_{3}(m_{a,i}), \, v_{b,i} = BiLSTM_{4}(m_{b,i}) \end{aligned}$$

12 $$\begin{aligned} v_{a, avg}= & {} \sum _{i=1}^{\ell _{a}} \frac{v_{a,i}}{\ell _{a}}, \, v_{b, avg} = \sum _{i=1}^{\ell _{b}} \frac{v_{b,i}}{\ell _{b}} \end{aligned}$$

13 $$\begin{aligned} v_{a, max}= & {} \max _{i=1}^{\ell _{a}}v_{a,i}, \, v_{b, max} = \max _{i=1}^{\ell _{b}}v_{b,i} \end{aligned}$$

14 $$\begin{aligned} v= & {} [v_{a,avg}, v_{a, max}, v_{b,avg}, v_{b,max}] \end{aligned}$$

### Active learning

The data trained in binary classification should be annotated by experienced experts with high cost. Active learning aims to use fewer samples to train without degrading much performance. which select a certain amount of samples by some strategies and sent them to professional experts for annotating. The whole process is iterative and the pseudo code is given in Algorithm 1. 
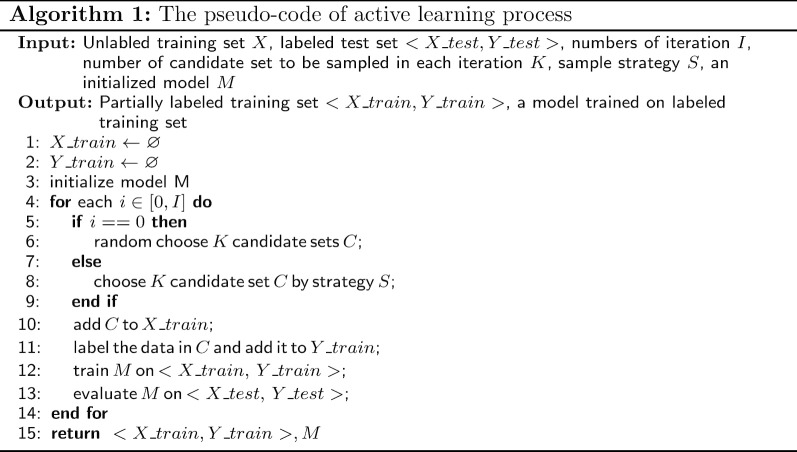
 The strategy used in active learning has a great importance to the final result. The main idea to select samples is based on the uncertainty about the prediction of the model. A variety of strategies have been proposed to calculate model prediction uncertainty, such as least confidence, margin sampling and shannon sampling, where margin sampling is same as least confidence in binary classification. Least confidence considers uncertainty as the difference of 1 and the maximum probability of the prediction. For example, in binary classification, the probability of the model prediction being 0 and 1 is $p_1$ and $p_2$, respectively, where $p_1 + p_2 = 1$. Then the uncertainty can be formulated as $1-max(p_1, p_2)$. If the probability on one label($p_1$ or $p_2$) is close to 1, then the sample with low uncertainty will not be selected. Shannon entropy use information entropy to evaluate uncertainty, which is high if the distribution of the model prediction is uniform and the sample should be selected. In our case, a set of candidate sets are selected in each iteration, and the uncertainty based on least confidence and entropy are described in formula 15–16. In which $p_{ij}$ refers the probability of *ith* non-standard indicator paired with *jth* candidate in $c^i$ predicted by the model. Because each candidate set has different length, we add a regularization term to represent the uncertainty of each candidate set.

15 $$\begin{aligned} c_{lc}^{i}= & {} \frac{\sum _{j=0}^{\ell _{c^i}}(1-max(p_{ij}(y=1|x), p_{ij}(y=0|x)))}{\ell _{c^{i}}} \end{aligned}$$

16 $$\begin{aligned} c_{entropy}^{i}= & {} -\frac{\sum _{j=0}^{\ell _{c^i}}p_{ij}(y=1|x) * \log {(p_{ij}(y=1|x))} + p_{ij}(y=0|x) * \log {( p_{ij}(y=0|x)})}{\ell _{c^{i}}} \end{aligned}$$

Besides least confidence and shannon entropy, we also propose geni index, a new uncertainty metric for sample selection. In decision tree, geni index is used to replace entropy because it is more efficient in computation and more suitable for binary classification. Gini index describes the probability of samples not belonging to the same category in two consecutive samples, which has the same property with shannon entropy that normal distribution would lead to a high value. Formula 17 shows the calculation of gini index of a candidate set $c^{i}$.

17 $$\begin{aligned} c^{i}_{gini\_index} = \frac{\sum _{j=0}^{\ell _{c^i}} 2*p_{ij}(y=1|x)*p_{ij}(y=0|x)}{\ell _{c^i}} \end{aligned}$$

**Fig. 2:**
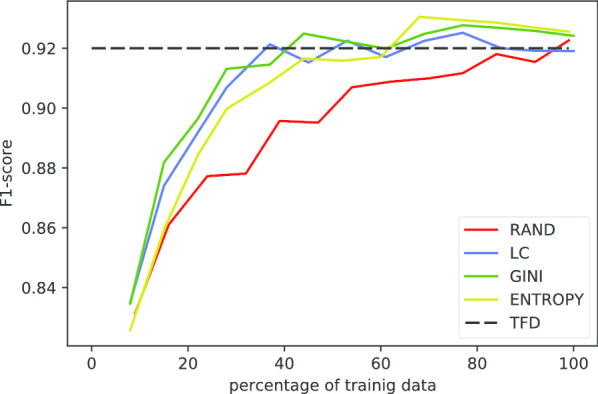
F1-score on test dataset with different amount of training data. RAND for random selection; LC for least confidence; GINI for gini index; ENTROPY for shannon entropy; TFD for trained with full data

**Fig. 3:**
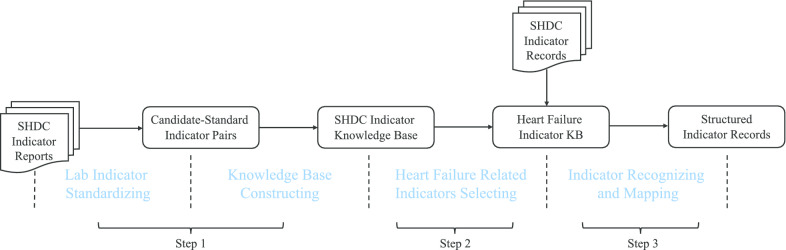
The process of the case study on heart failure

**Table 1:** Comparison of different recall model which only use name

Model	Recall	MRR
Edit distance	86.67	0.74
Bow (bag of words)	84.32	0.49
bm-25	90.10	0.35
tf-idf	92.50	0.82

MRR for mean reciprocal rank, which is the average of the reciprocal ranks of results for a sample of queries

**Table 2:** Comparison of different recall model which use both name and abbreviation

Model	Recall	MRR
Edit distance	91.83	0.79
Bow (bag of words)	89.78	0.53
bm-25	95.76	0.38
tf-idf	97.38	0.87

## Results

### Dataset

The dataset is collected from Shanghai Hospital Development Center. Among 38 hospitals, we choose 8 hospitals whose data are diverse and rich enough in synonymous indicators and types for our experiment. We extract two attributes of indicator from the dataset, namely name and abbreviation. Then a list of standard indicator names are selected and each standard indicator was annotated with some synonymous indicators by experts. The number of extracted indicators is 12,298, and the standard indicator list has 1970 items. As for the settings in our experiments, the recall number on indicator name $k_1$ is set to 15, and the number on indicator abbreviation $k_2$ is set to 5. In active learning, we select 100 candidate sets in each iteration. And we use SGD as optimizer for binary classification. For the evaluation of candidate selection task, Recall and MRR is used as metrics. We also use Precision, Recall and F1-score to measure the binary classification task. Finally, we split the dataset into train and test set randomly at the rate of 7:3.

### Candidate selection

We compare tf-idf with three other baselines:

- edit distance: a model measures the number of operations to transform one string to another.
- bow(bag of words): a basic model to represent text into vector for similarity calculation.
- bm-25: a baseline model in information retrieval.

The result is shown in Tables 1 and 2. In the result of Table 1, we only use the name information of indicator, Table 2 shows the result trained on both name and abbreviation. The Recall and MRR improves when abbreviation is added to each model, which shows abbreviation is an important feature in indicator term normalization. Beside, the tf-idf model outperforms all other models thus we choose tf-idf to select candidates of non-standard indicators.

### Binary classifications

For binary classification, we compare ESIM with Zhang’s methods [24] and another natural language inference method BIMPM [9]. Zhang utilizes DBSCAN for clustering and then uses stacking mechanism to learn a binary classification model on n_gram feature, it should be noticed that in addition to name and abbreviation of indicator, they also use the reference value as an important feature. BIMPM uses an advanced method of multi-perspective matching on LSTM for classification.

The results are shown in Table 3. As seen from the table, ESIM outperforms the other methods, with a Precision of 92.39$\%$ , a Recall of 91.78$\%$ and a F1-score of 92.08$\%$, respectively. In particular, ESIM is almost 10$\%$ higher on F1-score when compared with Zhang’s methods, which is because that ESIM can extract semantic information instead of handcraft feature used in Zhang’s methods. As for BIMPM, we suppose that its complicated structure is not suitable in the scenario of indicator normalization with shallow semantic information.

### Active learning

We empirically compare three active learning strategy mentioned above with a random baseline. The result is shown in Fig. 2. At the start of training, four strategies behaves similar because random selection is used in first iteration. All selection strategies performs better than random baseline after several rounds, where least confidence and gini index achieves a better F1-score in most rounds. Impressively, least confidence achieve the performance trained with full data using only 38$\%$ data, and gini index even performs better with 43$\%$ training data. After using 60$\%$ data for training, shannon entropy performs best in the four strategy. Note that gini index computes more efficient than shannon entropy.

**Fig. 4:**
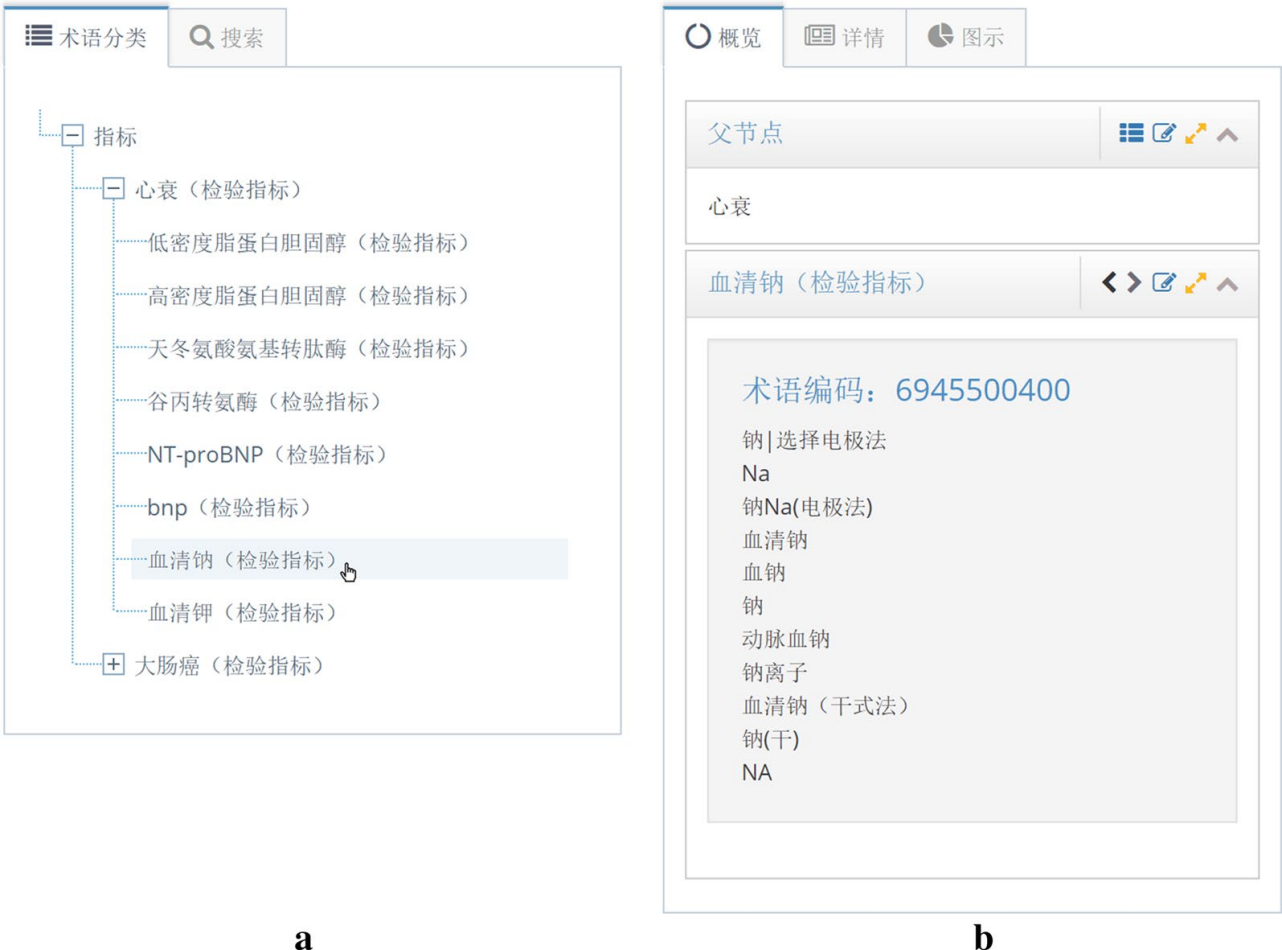
The screenshot of the heart failure indicator KB. **a** Lab indicators related to heart failure: Related indicators are picked out, and listed as standard names. The “serum sodium” is clicked this time. *b* Standard indicators and its synonyms: After clicked, the “serum sodium” and its synonymous names, which are all from the SHDC dataset and standardized by our proposed method, are displayed

**Fig. 5:**
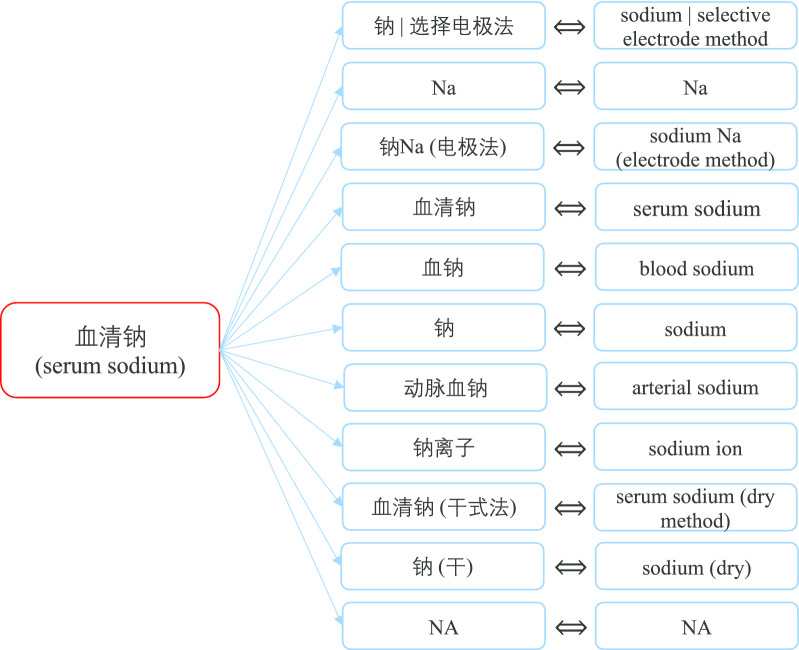
Different names of “serum sodium”

**Fig. 6:**
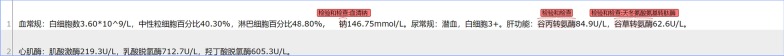
A demo of named entity recognition and mapping. All relevant indicators of heart failure are recognized. Since some indicators are synonymous names, they should be mapped to their standard names. That is, “sodium” is mapped to “serum sodium” and “serum glutamic-oxaloacetic transaminase” is mapped to its standard indicator named “glutamate oxaloacetate transaminase”

**Table 3:** Performance comparisons of binary classification

Methods	Precision	Recall	$\hbox {F}_1$-score
Zhang	88.38	79.89	83.53
BiMPM	83.07	90.13	86.19
ESIM	92.39	91.78	92.08

Zhang is the methods proposed in [24], a n-gram and stacking enhanced method

**Table 4:** Recall results of ”sodium ion”

Non standard indicator	Standard indicators		
			
		
		

## Discussion

We have experimentally evaluated our proposed standardization method in the section of Results. In this section, we further give a detailed case study on a real application, namely heart failure clinic analysis, to show the necessity of lab indicator standardization.

### Qualitative analysis

In order to better understand the performance of the proposed method, we manually analyze an indicator related to heart failure called “  (Serum sodium)” .

Since “  ” is a standard indicator selected by experts. We take its non-standard synonym “  ” as example. The first step is using recall model to select a candidate set. Then the binary classification model is used to get the standardization result. Tables 4 and 5 shows the result of recall model and classification model, respectively.

**Table 5:** Classification results of ”sodium ion”

Standard indicator	Non_standard indicator	Label	Predict
		1	1
		0	0
		0	0
		0	0
		0	0
		0	0
		0	0
		0	0
		0	0

The proposed method is able to produce presentable standardization results. It can be seen that the recall model successfully find a candidate set related to “  ”, in which contains many semantically similar indicators. The classification model is able to find the correct standard indicator and exclude all non-synonyms: “  ”, “  ”, “  ”, etc. Which is similar to the name of sodium ion in Chinese.

Nevertheless, our analysis also points out that the proposed standardization method has limitations. For candidate selection step, in Table 4, we observe that some irrelevant indicators such as “  ” and “  ” appear in the candidate set. This shouldn’t be a surprise since the recall model is based on character level and the indicators with overlapping characters have a certain chance of being clustered. Besides, such irrelevant negative samples will make the distribution of label in binary classification uneven, which brings challenges for further training process. It is remarkable that the recall method has included the groundtruth, regrettably at the expense of misjudging some irrelevant non-synonyms.

### Role of lab indicator standardization

The role of lab indicator standardization is then introduced. In academic research about association between use of statins and outcomes in heart failure, the lab indicator “serum sodium” is considered as a relevant factor. Before indicator standardization, only 50,094 records can be found out by searching name “  ” (serum sodium) in the SHDC dataset, which is obviously missing since there are more than 100 thousand inpatients in the research group and “serum sodium” is a common indicator in Shanghai hospitals. Observing the records in the dataset, we find that “serum sodium” is also described as “  ” (sodium), “  ” (ionized sodium). We then search the records using names supplemented by our algorithm and 150,000 records are associated.

So far, we can see that lab indicator standardization is helpful in the following procedures: data cleaning, data mining, text extracting and entity alignment. For data cleaning, standardization is its main step. For data mining and text extracting, more synonymous indicators standardized by our method, more records can be searched. For entity alignment, lab indicator standardization can be considered as an entity alignment problem.

### Application

The heart failure research is conducted on the sub-dataset from SHDC. The SHDC dataset contains EHRs from 2012 to 2016. Data specifications in different hospitals are all different so that expressions of the same item may be distinct. For example, one hospital uses lab indicators’ English names for abbreviations while another hospital uses inner numeric codes of the hospital. With the dataset, we use our algorithm to clean the data and preprocess the data according to the research goal and the guidance of specialist doctor for varies diseases. Figure 3 shows the process of the case study on heart failure. Generally, we can clean and preprocess lab indicator data in three steps. The first step is to construct the SHDC lab indicator KB. The second step is to pick out lab indicators related to special disease and construct the special disease lab indicator KB. The last step is to named entity recognize [32, 33] using the special disease indicator KB and turn text descriptions into structured data and map synonymous names into standard names.

The first step is mainly about our proposed method. A generic SHDC lab indicator KB is independent to special diseases or academic subjects and can be applied to all tasks based on the same dataset. As the generic KB can be used in lots of tasks, it can prevent duplication of data preprocessing and data cleaning while offering an easy-to-use standard for follow-up special disease researches. Specifically, we input lab indicator reports of the SHDC dataset into our algorithm and output candidate-standard indicator pairs into an easy-to-use KB. Each synonymous indicator is linked to its standard indicator and standard indicators are organized in the tree structure which is easy to view and mange.

The second step is the procedure of data selection. According to the requirement of a research, several related indicators are picked out into specific indicator KB. In this case, commonly used indicators in heart failure are put into a heart failure indicator KB. Figure 4 is a screenshot of specific indicator KB visual tool, lab indicators related to heart failure are listed on the left tree view, and they are all standard names. When we click on an indicator, all its synonyms will be displayed on the right part of the webpage. For instance, synonymous indicators of “serum sodium” are shown on the right part, which are standardized by our proposed method and preliminarily checked by medical students. Figure 5 is the detail results of serum sodium standardization.

The last step is the post-structure step. Each synonymous indicator is recognized using the heart failure indicator KB constructed above [32], and it is mapped to the standard indicator. Structured records are extracted effortlessly from the mapped entity. As shown in Fig. 6, all indicators related to heart failure are recognized, and the recognition is based on the KB constructed in the second step. The recognized indicators may already be standard names, or they may only be synonymous names. For synonymous names, we need to map them to their standard names. For example, “  ” (sodium) is mapped to “  ” (serum sodium) and “  ” (serum glutamic-oxaloacetic transaminase) is mapped to its standard indicator “  ” (aspartate aminotransferase).

## Conclusions and future work

In this paper, we propose an effective recall-and-classification structure based on active learning to standardize the lab indicators in SHDC. To decline the alignment scale, we test several classic text matching methods and finally utilize tf-idf to recall a candidate set for non-standard indicators. And to assure the accuracy of output, an enhanced sequential inference model(ESIM) is performed for binary classification. Experimental results show that ESIM can achieve an F1-score of 92.08$\%$ in the final binary classification. To reduce the cost of annotation, we add active learning on binary classification task. A new sample selection is proposed, which performs better than random baseline and could outperform the best score trained with full data with only 43$\%$ training data. Finally, a case study on heart failure clinic analysis shows that our proposed method is practical in an application with good performance.

As for future work, our proposed method includes synonyms at the expense of adding some irrelevant items in the case study, which suggests the possible research avenues that remain open. In the future, we also plan to optimize our proposed method to construct a lab indicator KB covering the whole data of Shanghai Hospital Development Center, and apply it to other clinic analyses.

## Data Availability

Our data is collected from the Shanghai Hospital Development Center, and the availability of these data were used under license for the current study. The Shanghai Hospital Development Center agreed to share these data for the purposes of this study only, therefore the data is not publicly available.

## Abbreviations

SHDC: Shanghai Hospital Development Center
EHRs: Electronic health records
KB: Knowledge base
KG: Knowledge graphs
RAND: Random based
ENTROPY: Shannon entropy
GINI: Gini index
LC: Least confidence
ESIM: Enhanced sequential inference model
BiLSTM: Bidirectional long short-term memory
BIMPM: Bilateral multi-perspective matching
MRR: Mean reciprocal rank
DBSCAN: Density-based spatial clustering of applications with noise

## References

[1] Arora S, Caughey MC, Misenheimer JA, Jones WM, Fish AC, Smith SC, Stouffer GA, Kaul P (2017). Elevated serum aspartate transaminase as a predictor of early mortality in patients with non-ST-segment elevation myocardial infarction. Circulation.

[2] Rong S, Niu X, Xiang EW, Wang H, Yang Q, Yu Y. A machine learning approach for instance matching based on similarity metrics. In: International semantic web conference. Springer, pp 460–475; 2012.

[3] Elmagarmid AK, Ipeirotis PG, Verykios VS (2006). Duplicate record detection: a survey. IEEE Trans Knowl Data Eng.

[4] Bilenko M, Mooney R, Cohen W, Ravikumar P, Fienberg S (2003). Adaptive name matching in information integration. IEEE Intell Syst.

[5] Suchanek FM, Abiteboul S, Senellart P (2011). Paris: probabilistic alignment of relations, instances, and schema. Proc VLDB Endow.

[6] Kong C, Gao M, Xu C, Fu Y, Qian W, Zhou A (2019). Enali: entity alignment across multiple heterogeneous data sources. Front Comput Sci.

[7] Hu W, Qu Y, Cheng G (2008). Matching large ontologies: a divide-and-conquer approach. Data Knowl Eng.

[8] Wang Z, Li J, Tang J. Boosting cross-lingual knowledge linking via concept annotation. In: Proceedings of the 23rd international joint conference on artificial intelligence. IJCAI, pp 2733–2739; 2013

[9] Wang X, Liu K, He S, Liu S, Zhang Y, Zhao J (2017). Multi-source knowledge bases entity alignment by leveraging semantic tags. Jisuanji Xuebao/Chin J Comput.

[10] Ruan T, Wang M, Sun J, Wang T, Zeng L, Yin Y, Gao J (2017). An automatic approach for constructing a knowledge base of symptoms in Chinese. J Biomed Semant.

[11] Zhang Y, Wang X, Lai S, He S, Liu K, Zhao J, Lv X, Sun M, Liu Z, Zhang M, Liun Y (2014). Ontology matching with word embeddings. Chinese computational linguistics and natural language processing based on naturally annotated big data.

[12] Kolyvakis P, Kalousis A, Kiritsis D. Deepalignment: unsupervised ontology matching with refined word vectors. In: Proceedings of the 2018 conference of the North American chapter of the association for computational linguistics: human language technologies, Volume 1 (Long Papers), vol. 1. ACL, pp. 787–798; 2018.

[13] Lei L, Zhou Y, Zhai J, Zhang L, Fang Z, He P, Gao J. An effective patient representation learning for time-series prediction tasks based on EHRS. In: IEEE international conference on bioinformatics and biomedicine, BIBM 2018, Madrid, Spain, December 3–6, 2018, pp 885–892; 2018.

[14] Kolyvakis P, Kalousis A, Smith B, Kiritsis D (2018). Biomedical ontology alignment: an approach based on representation learning. J Biomed Semant.

[15] Sun Z, Hu W, Zhang Q, Qu Y. Bootstrapping entity alignment with knowledge graph embedding. In: Proceedings of the twenty-seventh international joint conference on artificial intelligence (IJCAI), pp 4396–4402. IJCAI; 2018

[16] Trisedya BD, Qi J, Zhang R (2019). Entity alignment between knowledge graphs using attribute embeddings. Proc AAAI Conf Artif Intell.

[17] Cucerzan S. Large-scale named entity disambiguation based on Wikipedia data. In: Proceedings of the 2007 joint conference on empirical methods in natural language processing and computational natural language learning (EMNLP-CoNLL). ACL, pp 708–716; 2007

[18] Han X, Zhao J. Nlpr\_kbp in tac 2009 kbp track: a two-stage method to entity linking. In: TAC. Citeseer; 2009.

[19] Han X, Sun L, Zhao J. Collective entity linking in web text: a graph-based method. In: Proceedings of the 34th international ACM SIGIR conference on research and development in information retrieval. ACM, pp 765–774; 2011.

[20] Varma V, Pingali P, Katragadda R, Krishna S, Ganesh S, Sarvabhotla K, Garapati H, Gopisetty H, Reddy VB, Reddy K et al. Iiit hyderabad at tac 2009. In: TAC; 2009.

[21] Lehmann J, Monahan S, Nezda L, Jung A, Shi Y. LCCc approaches to knowledge base population at TAC 2010. In: TAC; 2010.

[22] Moreno JG, Besançon R, Beaumont R, D’hondt E, Ligozat A-L, Rosset S, Tannier X, Grau B. Combining word and entity embeddings for entity linking. In: European semantic web conference. Springer, pp 337–352; 2017.

[23] Shen W, Wang J, Luo P, Wang M. LINDEN: linking named entities with knowledge base via semantic knowledge. In: Proceedings of the 21st international conference on world wide web. ACM, pp 449–458; 2012.

[24] Zhang J, Wang Q, Zhang Z, Zhou Y, Ye Q, Zhang H, Qiu J, He P. An effective standardization method for the lab indicators in regional medical health platform using n-grams and stacking; 2019. 10.1109/BIBM.2018.8621274.

[25] Settles B. Active learning literature survey. Technical report, University of Wisconsin-Madison Department of Computer Sciences; 2009.

[26] Fu Y, Zhu X, Li B (2013). A survey on instance selection for active learning. Knowl Inf Syst.

[27] Shen Y, Yun H, Lipton ZC, Kronrod Y, Anandkumar A. Deep active learning for named entity recognition; 2017. arXiv:1707.05928.

[28] Joshi AJ, Porikli F, Papanikolopoulos N (2009) Multi-class active learning for image classification. In: 2009 IEEE conference on computer vision and pattern recognition. IEEE, pp 2372–2379.

[29] Hakkani-Tür D, Riccardi G, Gorin A. Active learning for automatic speech recognition. In: 2002 IEEE international conference on acoustics, speech, and signal processing, vol. 4. IEEE, p 3904; 2002.

[30] Devlin J, Chang M-W, Lee K, Toutanova K. Bert: pre-training of deep bidirectional transformers for language understanding; 2018. arXiv:1810.04805.

[31] Chen Q, Zhu X, Ling Z, Wei S, Jiang H, Inkpen D. Enhanced lstm for natural language inference; 2016. arXiv:1609.06038.

[32] Wang Q, Zhou Y, Ruan T, Gao D, Xia Y, He P (2019). Incorporating dictionaries into deep neural networks for the Chinese clinical named entity recognition. J Biomed Inform.

[33] Qiu J, Zhou Y, Wang Q, Ruan T, Gao J (2019). Chinese clinical named entity recognition using residual dilated convolutional neural network with conditional random field. IEEE Trans NanoBiosci.

